# Preliminary Investigation of the Effects of Rosemary Extract Supplementation on Milk Production and Rumen Fermentation in High-Producing Dairy Cows

**DOI:** 10.3390/antiox11091715

**Published:** 2022-08-30

**Authors:** Fanlin Kong, Shuo Wang, Dongwen Dai, Zhijun Cao, Yajing Wang, Shengli Li, Wei Wang

**Affiliations:** 1 Beijing Engineering Technology Research Center of Raw Milk Quality and Safety Control, The State Key Laboratory of Animal Nutrition, Department of Animal Nutrition and Feed Science, College of Animal Science and Technology, China Agricultural University, No. 2 Yuanmingyuan West Road, Haidian District, Beijing 100094, China; 2 College of Agriculture, Ningxia University, No. 489 West Helanshan Road, Yinchuan 750000, China

**Keywords:** high-producing, dairy cow, rosemary extract, milk production, rumen microbiota, antioxidant status

## Abstract

Rosemary extract (RE) has been used as an antioxidant in cosmetics and food additives, indicating its potential as a feed additive to improve adaptation in high-producing dairy cows. Here, we investigated the effects of RE supplementation on lactation performance and rumen fermentation in high-producing dairy cows. Thirty multiparous cows were blocked into 15 groups based on milk production and were randomly assigned to one of two treatments: 0 or 28 g/d of RE supplementation to the basic diet per cow. The experiment was conducted over a 74-day period, which included an initial two-week adaptation period. We observed significant increases in milk and milk lactose yields following RE supplementation. Somatic cell count tended to decrease by treatment. Additionally, superoxide dismutase concentration significantly increased and malonaldehyde level decreased after RE supplementation. Sequencing of 16S rRNA revealed that RE supplementation significantly affected the microbial composition and decreased the richness of the microbiota. Specifically, the abundance of the genus *Prevotella* was significantly decreased by RE supplementation and was correlated with volatile fatty acids in the Mantel test, whereas no significant correlation was found for other genera. Our findings provide fundamental information on the potential for RE as a feed additive for dairy cows to improve antioxidant status and enhance propionate generation.

## 1. Introduction

In recent years, the intensive systems used to raise dairy cows have maximized productivity and satisfied the demand for dairy products; however, this strategy requires the increased use of commercial feed as well as high stocking rates [1,2]. As a result, dairy cows fed high-concentrate diets are frequently exposed to metabolic stress, including oxidative stress, which is associated with deterioration in lactation performance and reproduction [3,4]. Furthermore, the dairy production chain is a potential transmission route for antibiotic residues, which encourages the spread of antibiotic resistance genes and antibiotic-resistant bacteria through manure [5]. To reduce the use of antibiotics and the cost of disease prevention, feed supplementation with diverse plant extracts may provide a relatively safe and healthy way to improve the antioxidant status of dairy cows due to the presence of prebiotics, such as polyphenols [6] and flavonoids [7].

Rosemary (*Rosmarinus officinalis* L.) belongs to the Lamiaceae family and is a perennial aromatic herb that originates from the Mediterranean area. It is now widely used in cosmetics, pharmaceuticals, and food additives, as well as cancer prevention due to the presence of carnosol, carnosic, and rosmarinic acid [8]. Rosemary has antibacterial [9], antioxidant [10], anticancer [11] and anti-inflammatory [12] properties;. these benefits expand its application to domestic animals, and many studies have shown that dietary supplementation with rosemary extract (RE) may improve growth performance and antioxidant status [13], regulate gut microbiota [14], modulate humoral immunity [15], alleviate heat stress [16], and enhance the quality of the product [17] in monogastric animals. Gladine et al., (2007) [18] demonstrated the antioxidant function of rosemary in ruminants by administering a single acute dose of rosemary to sheep using a duodenum T-shaped cannula. However, the presence of a rumen may influence the impact of RE. Further, feeding experiments found that adding rosemary and its by-products to the diet improved milk yield and quality in dairy sheep [19] and dairy goats [20], as well as meat quality [21]. The underlying mechanism may contribute to positive effects on ruminal fermentation [22] and ruminants may benefit from the antioxidant effects of rosemary [23]. Despite the dominant role of dairy cows compared with other ruminants in the dairy industry, very little is known about the application of RE in dairy cows.

High-production dairy cows typically experience high oxidative stress [24], which can be exacerbated under certain environmental, physiological, and dietary conditions [25]. Research on dairy cows has shown that the high-concentrate diets used to support the energy demands of high-production dairy cows cause remodeling of the microbial composition and function in the gastrointestinal tract of dairy cows [26,27,28]. For ruminants, the microbiota in the rumen serves as a bioreactor that enables dairy cows to obtain nutrients from human-indigestible plant matter [29], and multi-omics studies have revealed that the rumen microbiota is crucial for dairy cow performance [30]. Hence, caution should be exercised when using RE in dairy cow diets to enhance milk performance and oxidant status due to the potential antibacterial function of rosemary. However, the effect of RE on rumen fermentation is still not fully understood.

The objectives of this study were to: (1) explore the effect of RE supplementation on rumen fermentation and the rumen microbiota in high-production dairy cows, and (2) evaluate the possible benefits of RE on milk production and oxidant status. This preliminary investigation lays the foundation for a more comprehensive understanding of how the rumen responds to RE supplementation and provides a baseline for optimal dose investigation in the future.

## 2. Materials and Methods

### 2.1. Rosemary Extract

The RE was provided by the Hunan Zhizhiyuan Co., Ltd. (Changsha, China), and was obtained from rosemary leaves in the Hunan province of China. The RE was obtained using the solvent extraction method. The active components were quantified by UPLC-MS/MS. A 1 g sample was placed in 50 mL polypropylene centrifuge tubes, then 20 mL methanol was added. The mixture was ultrasonicated for 30 min and then centrifuged at 10,000 rpm for 5 min. An amount of 1 mL of the solution was transferred to a 100 mL volumetric flask, diluted with methanol to volume, and mixed. Finally, the sample was filtered through a PTFE syringe filter (0.22 µm, Ann Arbor, MI, USA) prior to UPLC-MS/MS analysis. Chromatographic separation was performed on a Waters Acquity H-class UPLC system with column oven temperature maintained at 40 °C, using an Acquity BEH C18 column (50 mm × 2.1 mm, 1.7 µm particle size) (Waters, MA, USA). The UPLC system was coupled to a Micromass Xevo TQ-S triple quadrupole mass spectrometer (Waters, Manchester, UK) fitted with an electrospray ionization source in negative mode (ESI-). Analytical standards of carnosic and rosmarinic acid were purchased from Sigma-Aldrich (St. Louis, MO, USA). The RE had the following functional composition: 42.28 g/kg carnosic and 1.59 g/kg rosmarinic acid. The UPLC-MS/MS chromatograms is presented in Supplementary Figure S1.

### 2.2. Animals and Treatments

The experiment was conducted at a commercial dairy farm in Beijing, China. Thirty multiparous Holstein cows averaging 63 ± 19 days in milk, 41 ± 3 kg/d of milk (mean ± SD) at the beginning of the experiment were sourced. The experiment included 14 days of adaptation followed by 60 days of formal experimentation. The animals were housed in a barn equipped with stalls and water bowls for free access to water. Dietary ingredients were mixed using a Labrador MT TMR mixer (STORTI Animal Husbandry Equipment Co., Ltd., Beijing, China) and offered as a total mixed ration three times daily at 08:00 h, 14:00 h, and 20:00 h for ad libitum intake. The basic diet was formulated using *Nutrient Requirements of Dairy Cattle* (2001) to meet the nutrient requirements (Supplementary Table S1).

Cows were blocked into 15 groups with two cows per group based on milk production, and cows within a block were randomly allocated to one of the two experimental diets: (1) cows in the CON group were only fed a basic diet, and (2) cows in the RE group were fed a basic diet supplemented with 28 g RE. The dose was determined according to dry matter intake (DMI) data from the feeding section of the dairy farm (24.19 kg/d) and the total antioxidant concentrate of RE (43.87 g/kg) such that the dietary concentrate of total antioxidants was 0.05 g/kg, which is in line with a previous study [31]. All cows were milked three times daily at 07:00, 13:00 and 19:00 h.

### 2.3. Sampling and Analysis

Refusals and offers of total mixed ration (TMR) were weighted daily for each group, and TMR samples were collected weekly and combined to determine the nutrient compositions according to Hao et al. [32]. Milk production by individual cows was recorded daily throughout the experiment. Individual milk samples from 07:00, 13:00, and 19:00 h at 0, 30, and 60 d were mixed at 4:4:3 to determine the milk compositions according to Kong et al. [33]. Fat-corrected milk (FCM) and energy-corrected milk (ECM) were calculated using the following equations according to a previous study [34]:

3.5% FCM=0.4324×milk production+16.216×milk fat productionECM=0.327×milk production+12.95×milk fat production+7.20×milk protein production

Blood samples from the coccygeal vein of each cow were collected 2 h before the morning feeding on day 60 using lithium heparin-containing vacuum tubes. Samples were centrifuged at 3000× *g* for 15 min at 4 °C to collect plasma. The samples were analyzed using a GF-D200. The total oxidative capacity (TAC), glutathione peroxidase (GSH-Px), superoxide dismutase (SOD), catalase (CAT) and malondialdehyde (MDA) concentrates were determined using commercial kits according to the manufacturer’s instructions (Beyotime biotechnology Ltd., Shanghai, China). CD4, CD8, immunoglobulin A (IgA), immunoglobulin G (IgG), and immunoglobulin M (IgM) levels were analyzed using ELISA kits (Abcam, Cambridge, UK) with a Thermo Multiskan Ascent (Thermo Fisher Scientific, Shanghai, China).

Rumen fluid was collected using an oral stomach tube before morning feeding on day 60 to determine the proportion of volatile fatty acids, pH, enzyme activity, and microbiota diversity. Briefly, rumen fluid was sampled from six cows in each group (CON and RE), which were close to average milk production. The tube was then cleaned with fresh warm water. Rumen fluid was collected after filtering using a four-layer cheesecloth. First, 150 mL of rumen fluid was discarded due to saliva contamination. Then, 50 mL of rumen fluid was collected and frozen at −20 °C for quantification of volatile fatty acids (VFAs) and enzyme activities. At the same time, the pH value of the fluid fraction was measured with a Sartorius PB-10 pH meter (Beijing Sartorius Instrument System Co., Ltd., Beijing, China). The rest of the fluid (approximately 2 mL) was frozen at −80 °C and used for microbial diversity analysis. The VFA proportion was determined using gas chromatography (Agilent 6890N, Agilent Technology, Inc, Beijing, China), as previously described [33]. The enzyme activities of rumen fluid, including endo-1,4-β-glucanase, exo-1,4-β-glucanase, β-1,3-1,4-glucanase, Xykabasem and acetylesterase were quantified using commercial kits from Suzhou Grace Biotechnology Co., Ltd. (Suzhou, China). The enzyme activities of endo-1,4-β-glucanase, exo-1,4-β-glucanase, and β-1,3-1,4-glucanase are presented as enzymatic units, with one enzymatic unit representing the amount of enzyme required to release 1 μg of reducing sugar at 37 °C. The enzyme activities of Xykabasem are presented as enzymatic units, with one enzymatic unit representing the amount of enzyme required to release 1 nmol of xylose at 40 °C. The enzyme activities of acetylesterase are presented as enzymatic units, with one enzymatic unit representing the amount of enzyme required to release 1 nmol of PNP at 40 °C.

The procedures for microbiota diversity analysis, including DNA extraction, amplification, sequencing, and quality-filtering, were conducted according to a previous study [33]. Briefly, DNA was extracted from 1 mL of rumen fluid using an E.Z.N.A^®^ Soil DNA Kit (Omega Bio-Tek, Norcross, GA, USA), and the concentration and purity were determined using a NanoDrop 2000 UV-vis spectrophotometer (Thermo Scientific, Wilmington, DE, USA). The V3-V4 of the bacterial 16S rRNA gene was amplified using primer pairs 338F and 806R and the amplification process was performed as previously described [31]. PCR products were extracted from a 2% agarose gel and purified using an AxyPrep DNA gel extraction kit (Axygen Biosciences, Union City, CA, USA). Finally, the purified amplicons were pooled in equimolar amounts and paired-end sequencing was performed using the Illumina MiSeq PE300 platform (Illumina, San Diego, CA, USA). Analysis of amplicon sequencing data was performed using quantitative insights into microbial ecology 2 (QIIME2, version 2022.2) [35]. All samples were subsampled to an equal library size of 27,804 sequences for downstream analyses. Taxonomic classification was performed using the SILVA database (v138), based on 99% sequence similarity [36]. All sequences were deposited in the NCBI Sequence Read Archive under the project numbers PRJNA699978 and SAMN28961691-SAMN28961700.

### 2.4. Statistical Analysis

The effects of RE on milk performance were analyzed using SAS software (version 9.4). A block experimental design was used for the time, group, and interaction effects between treatment and group according to the following model:

$$Y=\mu +T_{i}+G_{j}+TG_{ij}+B_{k}+\varepsilon_{ijkl}$$

where Y is the dependent variable, **μ** is the overall mean, **T_i_** is the time effect, **G_j_** is the group effect of RE supplementation, **TG_ij_** is the interaction effect between **T** and **G**, **B_k_** is the block effect, and **ε_ijkl_** is the random residual error. Cows were used as the experimental units. Differences were considered significant at *p* < 0.05.

The statistical significance of the effects of RE supplementation on plasma variables, rumen fermentation parameters, and enzyme activities were determined by one-way analysis of variance using SAS 9.4. Differences were considered statistically significant at *p* < 0.05.

Microbial diversity analyses were performed using the online Majorbio Cloud Platform (www.majorbio.com, accessed on 5 February 2022). Principal coordinated analysis (PCoA) and an analysis of similarities (ANOSIM) using Bray–Curtis matrices were performed to test the statistical differences between different groups. The effects of RE supplementation on α diversity indices and the relative abundance of genera were assessed using Welch’s *t*-test. All *p*-values were corrected using a false discovery rate of 0.05, and differences were considered significant at *p* < 0.05. To investigate the relationships between the detected microbiota and rumen fermentation parameters, a Mantel test was conducted between volatile fatty acids, enzyme activities, and significantly differentially detected genera using the LinkET package in R (Version 3.3.1). Spearman’s correlation analysis was conducted for significantly differentially detected genera.

## 3. Results

### 3.1. Milk Performance

The milk performance results are presented in Table 1. The average milk production in the CON group and the RE group was 40.94 kg/d and 41.76 kg/d, respectively. A significant interaction between the treatment and time was detected for milk production (*p* < 0.05). RE supplementation tended to increase milk production and ECM, and decrease the SCC in milk, although these changes were not statistically significant (0.05 < *p* < 0.10). The milk protein yield was significantly higher in the RE group than in the CON group (*p* < 0.05).

### 3.2. Plasma Variables

Plasma variables are presented in Table 2. The MDA concentration was significantly lower in the RE group than in the CON group (*p* < 0.05). Conversely, RE supplementation significantly improved the SOD concentration (*p* < 0.05). The concentrations of TAC, GSH-Px, immunoglobulin, CD4, and CD8 were not affected by RE supplementation (*p* > 0.05).

### 3.3. Rumen Fermentation Parameters

Analysis of rumen fermentation parameters from the CON and RE groups are shown in Table 3. The pH value was not affected by RE supplementation (*p* > 0.05). VFA and individual VFA concentrations, except for propionate, were similar between the two groups (*p* > 0.05). The propionate concentration was significantly higher in the RE group compared to the CON group (*p* < 0.05), and RE supplementation tended to increase the molar proportion of propionate, although this increase was not statistically significant (0.05 < *p* < 0.10).

### 3.4. Rumen Enzyme Activities

Table 4 shows the results obtained from enzyme activities. Rumen fluid collected from the RE group exhibited significantly increased acetylesterase enzymatic activity compared to that from the CON group (*p* < 0.05). Conversely, dietary RE supplementation did not alter the enzyme activities of endo-1,4-β-glucanase, exo-1,4-β-glucanase, β-1,3-1,4-glucanase, or Xykabasem (*p* > 0.05).

### 3.5. Rumen Microbiota Analysis

#### 3.5.1. Microbiota α Diversity and β Diversity

**Figure 1:**
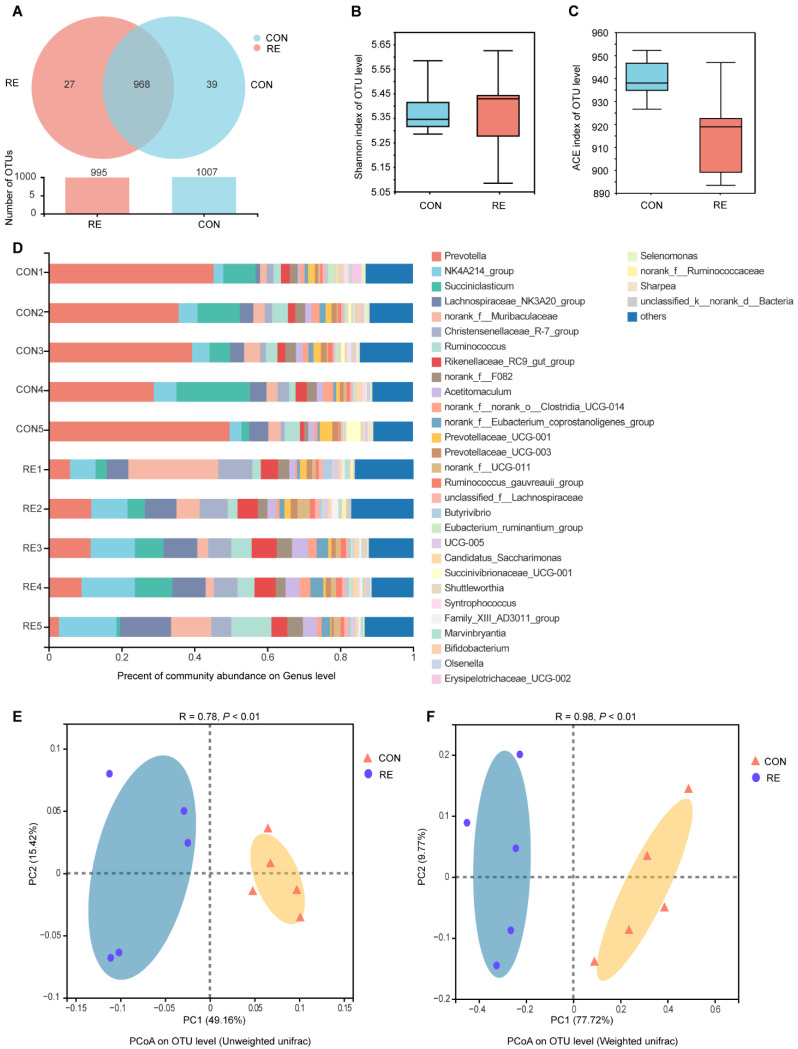
Effects of rosemary extract supplementation on rumen microbiota diversity in high-producing Holstein dairy cows. (**A**) Venn diagram showing the common and unique OTUs between the RE and CON groups; (**B**) Shannon index of the OTU level; (**C**) ACE index of the OTU level; (**D**) Composition of the rumen microbiota at the genus level; (**E**) Beta diversity of the OTU level based on unweighted UniFrac; (**F**) Beta diversity on the OTU level based on weighted UniFrac. RE, rosemary extract treatment group; CON, the control group.

The number of operational taxonomic units (OTUs) in the RE and CON groups were 995 and 1007, respectively (Figure 1A). We detected 27 unique OTUs in the RE group, 39 unique OTUs in the CON group, and 968 OTUs shared between the two groups (Figure 1A). The α diversity data presented in Figure 1B,C show that the Shannon index was not affected by RE supplementation (*p* > 0.05), whereas the ACE index tended to decrease (0.05 < *p* < 0.10). The community bar plot in Figure 1D demonstrates that *Prevotella* was the dominant genus in samples from the CON group. Furthermore, the results of PCoA indicated that the RE group exhibited a significant divergence from the CON group (ANOSIM, *p* < 0.05).

#### 3.5.2. Differentially Detected Genera

**Figure 2:**
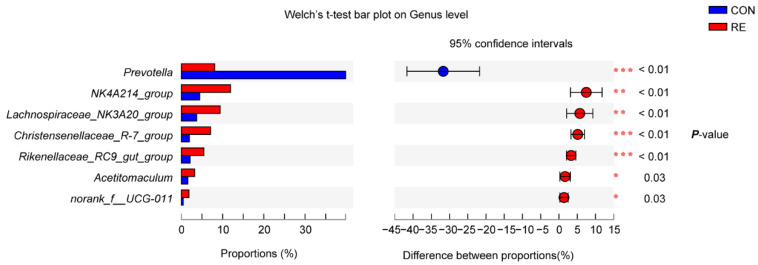
The relative abundances of significantly differentially detected genera between control and rosemary extract groups. Significantly differentially detected genera with relative abundances ≥1 are shown. Welch’s T-test was used to determine statistical significance. *, *p* ≤ 0.05; **, *p* ≤ 0.01; ***, *p* ≤ 0.001. RE, rosemary extract treatment group; CON, control group.

The relative abundances of significantly differentially detected genera are presented in Figure 2. Only genera with a relative abundance above 1% were included. The abundance of *Prevotella* was significantly lower in the RE group than in the CON group (*p* < 0.05). Conversely, the abundances of *NK4A214*_*group*, *Lachnospiraceae*_*NK3A20_group*, *Christensenellaceae*_*R-7_group*, *Rikenellaceae*_*RC9_gut_group*, *Acetitomaculum*, and *norank_f_UCG-011* were significantly increased following RE supplementation (*p* < 0.05).

### 3.6. Correlation Analysis between Rumen Fermentation Parameters, Enzyme Activities, and Significantly Differentially Detected Genera

**Figure 3:**
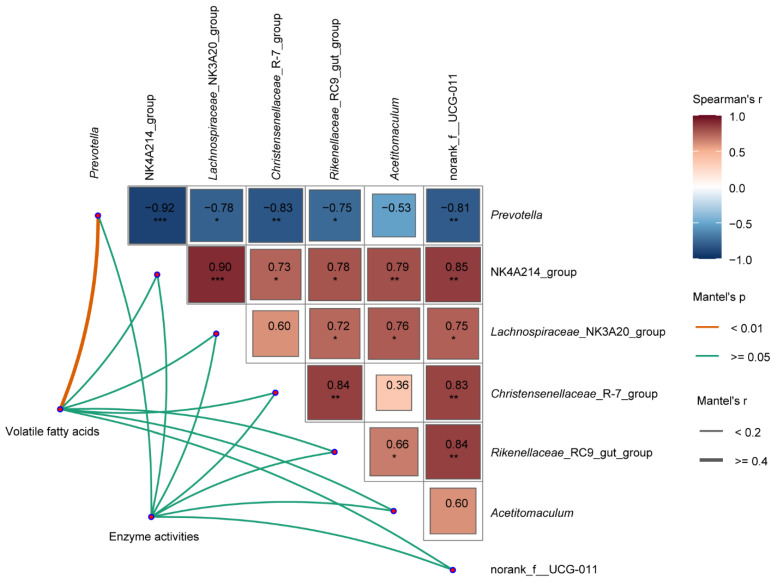
Mantel’s and Spearman’s correlation analyses. Pairwise comparisons of significantly differentially detected genera using Welch’s *t*-tests are shown, with a color gradient representing Spearman’s correlation coefficients. The asterisk indicates *p* value (*, *p* ≤ 0.05; **, *p* ≤ 0.01; ***, *p* ≤ 0.001). The correlation between volatile fatty acids or enzyme activities with each genus were determined using Mantel’s correlation tests. Edge width corresponds to the Mantel’s r statistic for the corresponding distance correlations, and edge color indicates the statical significance based on 999 permutations.

To investigate the potential impacts of the altered microbiota, we correlated the distance-corrected dissimilarities of VFAs and enzyme activities with the significantly differentially detected genera. *Prevotella* exhibited the strongest correlation with VFAs (*p* < 0.01, r ≥ 0.4), whereas no significant correlation was found for other genera (*p* ≥ 0.05, r < 0.2, Figure 3).

## 4. Discussion

Plant extracts are mixtures of different chemical substances, some of which contain beneficial properties. While the action of plant extracts is generally considered positive overall, the effectiveness of RE on dairy cow production had not previously been established. In this study, we fed healthy Holstein dairy cows with RE-supplemented feed to investigate the effects of RE supplementation on milk performance, antioxidant status, and the rumen microbiota. Our results demonstrated that RE supplementation improved milk performance, including milk production and lactose and protein yields.

RE and its by-products (distillation residues) are frequently used in dairy ewes; Chiofalo et al. found that 1200 mg RE per day improved milk fat, milk protein and milk lactose production [31]. Smeti et al. fed rosemary leaves (60 g/kg based on concentrate weight) to dairy goats and found that daily milk production was increased with the same feed intake [37]. Kholif et al. reported that the inclusion of 10 g rosemary whole plant daily in the diet of dairy goats did not affect feed intake and improved milk production consistent with our results [22]. This increase in milk production may be due to an increase in lactose yield. Lactose is the main solid in bovine milk and is responsible for the osmotic equilibrium between the blood and alveolar lumen in the mammary gland [38]. Although different forms (distillation residues, essential oils, rosemary extract, and raw rosemary) and doses of rosemary were used in these studies, the combined results demonstrate the positive effects of rosemary supplementation on milk production.

Dairy cows are most susceptible to metabolic diseases during the peak lactating period because reactive oxygen species are produced as by-products of enhanced cellular metabolism [4,39,40]. In general, the major effective components in RE are carnosol, carnosic acid, and rosmarinic acid, of which carnosic acid is the most abundant and has among the highest antioxidant activities [41,42]. Jordan et al. demonstrated the antioxidant efficacy of dietary carnosic and carnosol supplementation after ruminal fermentation in lambs [43]. MDA is a decomposition product of lipid hydroperoxides and is used as an indicator of oxidative damage to cells and tissues. In our study, RE supplementation decreased the MDA concentration and increased the SOD concentration, indicating that RE improved the oxidant status of dairy cows. Michelotti et al. directly infused carnosic into the veins of transition dairy cows and observed a positive effect on inflammation and oxidative stress biomarkers [44]; the underlying mechanism may involve the activation of the PI3K/AKT/Nrf2 signaling pathway [45,46]. Furthermore, our results indicated that RE supplementation may decrease the SCC in milk. This may have important implications for mastitis, which is defined as the inflammation of the mammary gland leading to the recruitment of polymorphonuclear leukocytes and typically increases the SCC in milk [47]. Dairy cows in the two groups in our study were assumed to be healthy cows without mastitis (i.e., SCC < 200,000 cells/mL) [48]. Our results indicated that RE may have the potential to improve mammary gland conditions.

Plant extracts are potential rumen modifiers because of their antimicrobial activity against microorganisms [49]. The wide application of plant extracts for reducing ruminal methane emissions supports their role as rumen modifiers [50]. Therefore, it is essential to evaluate the effects of RE on rumen functions. In our study, RE supplementation altered the rumen microbiota, resulting in different distributions of enzyme activities and VFAs. The α diversity indices revealed lower richness in cows with RE supplementation; lower richness has been considered a characteristic of higher VFA production, higher milk yield, and lower milk fat production in dairy cows [51], as well as higher feed efficiency in beef cattle [52]. This relationship may be explained by the more diverse use of nutrients by inefficient microorganisms in the rumen.

We detected an increase in the propionate concentration after RE supplementation; however, consistent with the findings of previous studies [22,53], the average pH value did not change in our study. This suggests that RE supplementation may enhance energy utilization by increasing the output of propionate, which is a precursor of glucose. Individual dry matter intake data was not collected due to equipment limitations, but the average daily dry matter intake between the two groups was similar. Hence, the increased ECM output (1.73 kg/d) observed in our study indicated an increase in energy utilization. A meta-analysis of the effects of essential oils on rumen fermentation showed that essential oils enriched in carnosic acid and carvacrol reduced acetate concentrations and increased propionate concentrations [54], consistent with our results. Furthermore, the increasing propionate concentrate points toward the potential of RE supplementation to lower the production of hydrogen, the electron donor in methanogenesis. However, few studies have been conducted on the methane emissions after RE supplementation in dairy cows and different forms of rosemary had different impacts on rumen methanogens in sheep and goats [55,56]. Thus, further studies are needed to investigate the role of RE in methane emissions.

An unchanged rumen fluid pH value reflects the stability of the rumen; however, our β diversity analysis indicated that RE supplementation influenced the microbiota. Carnosic acid and carnosol displayed high antibacterial activity in vitro, whereas rosmarinic acid did not display any activity against specific microorganisms [57]. To our knowledge, this is the first study to evaluate changes in the microbiota using 16S rRNA sequencing. In our study, *Prevotella* was the most significantly differentially detected genus between the CON and RE groups. Consistent with our results, dietary supplementation with rosemary in sheep also decreased the abundance of the genus *Prevotella* [55]. This genus utilizes starch and protein to produce succinate and acetate and is one of the most abundant core genera in the rumen of dairy cows [58]. The Mantel test confirmed the critical role of *Prevotella* in VFA generation in the rumen. Indeed, *Prevotella* was considered a biomarker for dairy cows with high milk protein yield [59]. Considering the decrease in the abundance of *Prevotella* following RE supplementation in our study, the concurrent increase in milk protein yield may be attributed to the digestion of rumen undegraded proteins in the small intestine and compensatory digestion by other genera, as microorganisms in the rumen have been shown to adapt to the antimicrobial activity of rosemary within 30 days [54].

The other significantly differentially detected genera from our study were more abundant in the RE group than in the CON group. These results may explain the stable rumen fermentation. Martinez et al. found that the genus *NK4A214* is associated with genetic lines selected by milk persistence, although the mechanism remains unclear [60]. *Christensenellaceae R-7 group* are gram-negative rod-shaped cells [61] and metagenomic analysis has revealed their role in producing acetate via hemicellulose degradation in the rumen. Gram-negative bacteria are likely to be less sensitive to rosemary essential oil; therefore, the interactions between other microorganisms may contribute to this change. A previous study found that loose rosemary leaves decreased the abundance of archaea [55], and another study found that essential oil containing 25.71% carnosic acid and 22.86% carvacrol depressed the rumen protozoa population [54]. Therefore, metagenomic analyses may provide a better understanding of the effects of RE on the microbiota. *Rikenellaceae* RC9 gut group belongs to the Rikenellaceae family, which typically ferments carbohydrates or proteins [62]. Previous studies have shown that the relative abundance of this genus decreased by 69.8% when the content of neutral detergent fiber in the diet was reduced from 39.07 to 30.9%, indicating that this genus may be responsible for fiber degradation. Finally, as reported in a previous study, *Acetitomaculum* mainly exists in the rumen of cows fed a high-concentrate diet and can utilize monosaccharides to produce acetate [63]. Therefore, compensatory increases from several high-abundance genera may contribute to the steady-state of the rumen in RE supplemented dairy cows.

## 5. Conclusions

Under the experimental conditions applied, RE supplementation improved milk yield and milk lactose yield, possibly by enhancing propionate production in the rumen and improving the antioxidant status in high-producing dairy cows. Moreover, RE supplementation reduced the relative abundance of *Prevotella* and increased the abundance of several genera associated with nutrient degradation. Notably, the pH value of the rumen was unaffected by RE supplementation, indicating that the steady-state of the rumen was not affected. Our study provides a preliminary investigation of RE on dairy cows and demonstrates the benefits of RE on milk performance and antioxidant status with no adverse effect on rumen fermentation. In this study, the recommended dose of RE was determined based on previous work on dairy ewes. The optimal dose of RE and the effects of RE on protozoa, fungi, and archaea must be further investigated at different stages, especially in transition dairy cows. Furthermore, data regarding DMI should be acquired to investigate feed efficiency. Overall, our findings demonstrate that RE supplementation has the potential to improve milk performance while maintaining rumen fermentation in high-producing dairy cows and is a candidate to replace antibiotics.

## Figures and Tables

**Table 1:** Effects of rosemary extract supplementation on milk performance in high-producing Holstein dairy cows.

Items	Groups		SEM	*p*-Value		
	CON	RE		Group	Time	Group∗Time
Milk production, kg/d	40.94	41.76	0.204	0.05	0.11	0.03
FCM yield, kg/d	47.36	49.52	1.571	0.39	0.06	0.16
ECM yield, kg/d	43.78	45.51	0.444	0.05	0.10	0.10
Milk fat, %	4.46	4.65	0.314	0.59	0.41	0.91
Milk protein, %	3.35	3.40	0.021	0.13	0.62	0.49
Milk lactose, %	5.03	5.02	0.046	0.85	0.07	0.62
SCC, ×1000/mL	152.60	126.42	8.415	0.09	0.96	0.75
Milk fat yield, kg/d	1.83	1.94	0.186	0.70	0.13	0.32
Milk protein yield, kg/d	1.37	1.42	0.004	<0.01	0.54	0.20
Milk lactose yield, kg/d	2.06	2.10	0.023	0.33	0.08	0.06

RE, rosemary extract treatment group; CON, control group; SEM, standard error; FCM, fat-corrected milk; ECM, energy-corrected milk; SCC, somatic cell count.

**Table 2:** Effects of rosemary extract supplementation on plasma antioxidant and immune indices in high-producing Holstein dairy cows.

Items	Groups		SEM	*p*-Value
	CON	RE		
TAC, U/mL	11.91	12.51	0.708	0.68
GSH-Px, mmol/L	13.34	16.75	1.144	0.14
MDA, mmol/mL	1.64	1.24	0.048	<0.01
SOD, U/mL	136.97	164.60	3.871	<0.01
IgA, μg/mL	24.40	24.48	0.375	0.92
IgG, mg/mL	430.15	417.15	6.674	0.34
IgM, mg/mL	12.90	12.26	0.202	0.12
CD4, ng/mL	6.09	5.96	0.317	0.85
CD8, ng/mL	39.94	39.44	0.498	0.63

RE, rosemary extract treatment group; CON, control group; SEM, standard error; TAC, total oxidative capacity; GSH-Px, glutathione peroxidase; MDA, malonaldehyde; SOD, superoxide dismutase; IgA, immunoglobulin A; IgG, immunoglobulin G; IgM, immunoglobulin M.

**Table 3:** Effects of rosemary extract supplementation on rumen volatile fatty acid characteristics in high-producing Holstein dairy cows.

Items	Groups		SEM	*p*-Value
	CON	RE		
pH	6.08	6.06	0.064	0.46
Concentration, mmol/L				
TVFA	110.50	116.63	2.138	0.16
Acetate	67.46	69.11	1.029	0.46
Propionate	29.11	33.02	0.967	0.03
Butyrate	10.31	10.76	0.449	0.65
Valerate	1.80	1.67	0.079	0.45
Branched-chain fatty acid	1.82	2.07	0.101	0.23
Molar proportion, %				
Acetate	61.14	59.25	0.594	0.11
Propionate	26.30	28.35	0.564	0.06
Butyrate	9.31	9.19	0.263	0.84
Valerate	1.62	1.43	0.065	0.17
Branched-chain fatty acid	1.64	1.78	0.074	0.37
Ratio				
Acetate: Propionate	2.34	2.09	0.068	0.07

RE, rosemary extract treatment group; CON, control group; SEM, standard error; TVFA, total volatile fatty acid.

**Table 4:** Effects of rosemary extract supplementation on enzyme activities of rumen fluid in high-producing Holstein dairy cows.

Items	Groups		SEM	*p*-Value
	CON	RE		
endo-1,4-β-glucanase, U/mL	388.83	311.46	30.352	0.22
exo-1,4-β-glucanase, U/mL	287.92	368.29	38.722	0.33
β-1,3-1,4-glucanase, U/mL	11.28	13.56	1.374	0.44
Xykabasem, U/mL	29.03	33.38	2.446	0.42
acetylesterase, U/mL	0.37	0.54	0.036	0.01

RE, rosemary extract treatment group; CON, control group; SEM, standard error.

## Data Availability

The raw data were deposited in the NCBI Sequence Read Archive (SRA) database (Accession Numbers: SAMN28961691-SAMN28961700).

## Supplementary Materials

The following supporting information can be downloaded at: https://www.mdpi.com/article/10.3390/antiox11091715/s1, Figure S1: UPLC-MS/MS chromatograms of rosemary extract, Table S1: Ingredients and nutrient compositions of basic diet.
