# Supplementary material for: Preliminary Investigation of the Effects of Rosemary Extract Supplementation on Milk Production and Rumen Fermentation in High-Producing Dairy Cows
Source: Antioxidants (Basel). 2022 Aug 30;11(9):1715. doi: 10.3390/antiox11091715 (PMC9495500; doi:10.3390/antiox11091715)

antioxidants

MDPI

Article

Preliminary investigation of the effects of Rosemary extract supplementation on rumen fermentation and milk performance in high-producing dairy cows

Fanlin Kong [1], Shuo Wang [1], Dongwen Dai [2], Zhijun Cao [1], Yajing Wang [1], Shengli Li [1,]* and Wei Wang [1,]*

[1] Beijing Engineering Technology Research Center of Raw Milk Quality and Safety Control, The State Key Laboratory of Animal Nutrition, Department of Animal Nutrition and Feed Science, College of Animal Science and Technology, China Agricultural University, No. 2 Yuanmingyuan West Road, Haidian District, Beijing 100094, China

[2] College of Agriculture, Ningxia University, No. 489 West Helanshan Road, Yinchuan 750000, China

* Correspondence: lishengli@cau.edu.cn (S.L.); wei.wang@cau.edu.cn (W.W.); Tel.: +86-010-62731254 (S.L.); +86-010-62733789 (W.W.)

Supplementary Figure S1. UPLC-MS/MS chromatograms of rosemary extract.

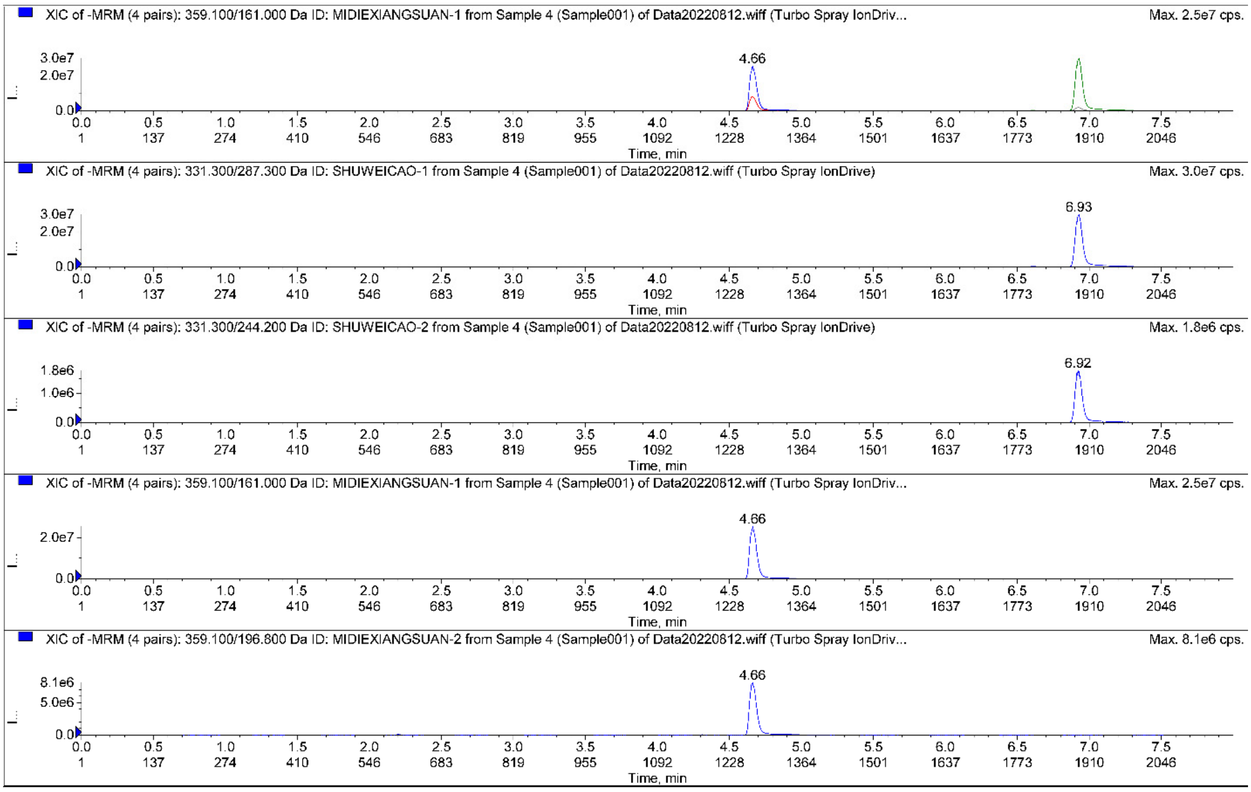

Supplement: Supplementary file 1 [file antioxidants-11-01715-s001.zip › Supplementary Figure S1.pdf]
