# Supplementary material for: Preliminary Investigation of the Effects of Rosemary Extract Supplementation on Milk Production and Rumen Fermentation in High-Producing Dairy Cows
Source: Antioxidants (Basel). 2022 Aug 30;11(9):1715. doi: 10.3390/antiox11091715 (PMC9495500; doi:10.3390/antiox11091715)
Supplement: Supplementary file 1 [file antioxidants-11-01715-s001.zip › Supplementary Table S1.pdf]

antioxidants

MDPI

Article

Preliminary investigation of the effects of Rosemary extract supplementation on rumen fermentation and milk performance in high-producing dairy cows

Fanlin Kong [1], Shuo Wang [1], Dongwen Dai [2], Zhijun Cao [1], Yajing Wang [1], Shengli Li [1,*] and Wei Wang [1,*]

[1] Beijing Engineering Technology Research Center of Raw Milk Quality and Safety Control, The State Key Laboratory of Animal Nutrition, Department of Animal Nutrition and Feed Science, College of Animal Science and Technology, China Agricultural University, No. 2 Yuanmingyuan West Road, Haidian District, Beijing 100094, China

[2] College of Agriculture, Ningxia University, No. 489 West Helanshan Road, Yinchuan 750000, China

* Correspondence: lishengli@cau.edu.cn (S.L.); wei.wang@cau.edu.cn (W.W.); Tel.: +86-010-62731254 (S.L.); +86-010-62733789 (W.W.)

Supplementary Table S1. Ingredients and nutrient compositions of basic diet.

Items	Value
Diet ingredients, %	
Corn silage	55.80
Oat hay	2.50
Alfalfa	6.30
Distillers dried grains with soluble	1.60
Flaked corn	5.20
Soybean husk	1.90
Cottonseed	1.60
Fatty powder	1.27
Extruded soybean	1.88
Corn	6.22
Bran	2.47
Soybean meal	6.77
Rapseed meal	0.94
Cottonseed meal	2.51
Pre-mix [1]	3.14
Nutrient level, of dry matter	
Net energy of lactation [2], Mcal/kg	1.67
Crude protein, %	16.60
Ether extract, %	5.77
Neutral detergent fiber, %	33.20
Acid detergent fiber, %	19.20
Ash, %	8.71
Ca, %	0.96
P, %	0.45

[1] Formulated to provide (per kg of dry matter): 250,000 IU of vitamin A, 50,000 IU of vitamin D_3, 1100 IU of vitamin E, 250 mg of Cu, 500 mg of Mn, 1,000 mg of Zn, 20 mg of Se, 40 mg of I, 24 mg of Co

[2] Net energy for lactation was estimated using NRC (2001) model.
